# Scalable Registration of Single Quantum Emitters within Solid Immersion Lenses through Femtosecond Laser Writing

**DOI:** 10.1021/acs.nanolett.5c01325

**Published:** 2025-06-10

**Authors:** Alexander R. Jones, Xingrui Cheng, Shravan Kumar Parthasarathy, Muhammad Junaid Arshad, Pasquale Cilibrizzi, Roland Nagy, Patrick Salter, Jason Smith, Cristian Bonato, Christiaan Bekker

**Affiliations:** 1 Institute of Photonics and Quantum Sciences, SUPA, 3120 Heriot-Watt University, Edinburgh EH14 4AS, UK; 2 Department of Engineering Science, 6396 University of Oxford, Parks Road, Oxford OX1 3PJ, UK; 3 Department of Materials, University of Oxford, Parks Road, Oxford OX1 3PH, UK; 4 Institute of Applied Quantum Technologies, 98884 Friedrich-Alexander-University Erlangen-Nürnberg, 91052 Erlangen, Germany; 5 Fraunhofer Institute for Integrated Systems and Device Technology (IISB), 91058 Erlangen, Germany

**Keywords:** Color Centers, Femtosecond Laser Writing, Silicon Carbide, Scalable Quantum Devices, Qubits

## Abstract

The precise registration of solid-state quantum emitters to photonic structures is a major technological challenge for fundamental research (e.g. in cavity quantum electrodynamics) and applications to quantum technology. Standard approaches include the complex multistep fabrication of photonic structures on pre-existing emitters, both registered within a grid of lithographically-defined markers. Here, we demonstrate a marker-free, femtosecond laser writing technique to generate individual quantum emitters within photonic structures. Characterization of 28 defect centers, laser-written at the centers of pre-existing solid immersion lens structures, showed offsets relative to the photonic structure's center of 260 nm in the *x*-direction and 60 nm in the *y*-direction, with standard deviations of ± 170 and ± 90 nm, respectively, resulting in an average 4.5 times enhancement of the optical collection efficiency. This method is scalable for developing integrated quantum devices using spin-photon interfaces in silicon carbide and is easily extendable to other materials.

## Graphical abstract

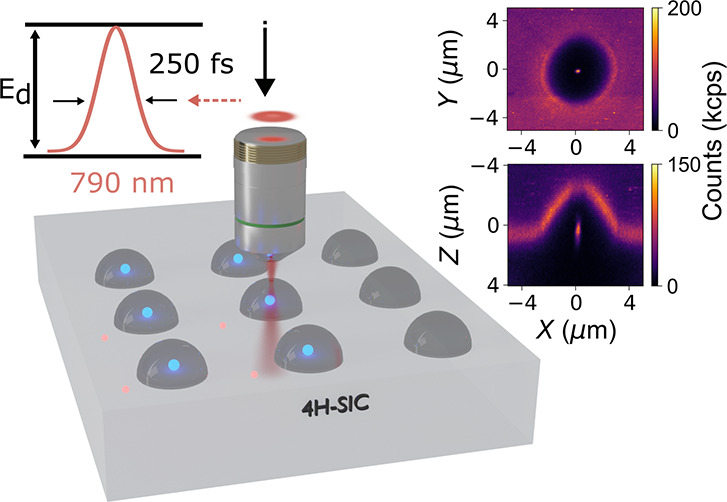

Color centers in the solid state, such as optically-active point defects and impurities, are among the most prominent systems for quantum technology.^1^ Spin-photon interfaces associated to color centers in diamond,^2–4^ silicon carbide (SiC)^5–9^ and silicon,^10,11^ along with single rare-earth ion dopants in crystals,^12^ have been used in pioneering demonstrations of long-distance quantum networks. The nitrogen-vacancy center in diamond, as well as other defects in SiC^13–16^ and hexagonal boron nitride,^17–20^ are used in a variety of quantum sensing applications, ranging from fundamental physics to healthcare.^21^

Enhancing photon collection efficiency through tailored photonic structures is critical for optically-read-out spin qubits, as it directly impacts spin-photon interface efficiency in quantum networks and sensitivity in quantum sensing.^22,23^ This has been achieved, for example, with simple structures that minimize the effect of total internal reflection inside a high-index material, such as solid immersion lenses (SILs),^24–28^ or light guiding structures such as waveguides.^29–34^ The alternative is to employ a microcavity, such as a nanopillar,^35–39^ bullseye cavity^40^ or photonic crystal,^41,42^ to enhance light-matter interaction, maximize the fraction of emission into the coherent zero-phonon line, and increase optical extraction efficiency.^43,44^

A significant challenge in scaling the integration of quantum emitters into photonic structures is registering them to the location that provides the greatest optical enhancement. Depending on the structure, this region can range from ∼1 *μ*m^3^ in SILs to ∼10 nm^3^ for photonic crystal cavities. One approach to registering single quantum emitters in photonic structures consists of mapping the position of pre-existing emitters against a marker array, which can be aligned to during photonic structure fabrication.^45–47^ This procedure could provide accuracy down to a few nanometers^45^ and enables pre-selection of emitters with optimal properties for integration. However, it is extremely time-consuming and not easily scalable to large arrays.

A second possibility is to implant the ion species required to create the emitter into existing photonic structures, for example, through the use of focused ion beams.^40,48–50^ This technique is very effective but can only be used to create shallow emitters (depths of ≲1 *μ*m), as the lateral accuracy of implanted ion placement is similar to the implantation depth. Higher energy ions for deep implantation can degrade emitter quality.^51–53^

Recently, the generation of quantum emitters by laser-writing has received increasing attention. This technique exploits highly energetic carriers, created either by below- ^33,54–58^ or above-bandgap^59^ illumination with a high-power femtosecond laser to initiate an avalanche ionization process. Above-bandgap illumination has been used to create quantum emitters in SiC nanophotonic structures, and femtosecond NIR laser pulses have been utilized to generate ensembles of quantum emitters in diamond nano-cavities.^40^

Here, we create single quantum emitters registered to SILs by direct below-bandgap femtosecond laser writing. Femtosecond laser writing enables us to create intrinsic point defects based on vacancies directly in the focal region of the SILs. This method does not require alignment to markers and can be fast, making it compatible with wafer-scale processing. The photonic structure itself can enhance the laser-writing field, potentially removing the need for any registration effort. By using the focusing effects of the lens, weaker laser pulse energies can be used to generate emitters. In contrast to ion implantation and above-bandgap illumination, direct below-bandgap femtosecond laser writing enables the creation of single quantum emitters deeper within micron-scale structures, where they are less sensitive to surface noise and typically exhibit better quantum coherence properties.

We focus on quantum emitters in SiC, a material that uniquely combines spin-photon interfaces^5,6,8^ possessing long spin coherence times^7,60^ with integrated photonic^29,61–63^ and microelectronic^64,65^ functionalities. Our approach readily adapts to alternative materials, such as diamond.^24,46,66^

The experiment was conducted using commercial 4H-SiC material (Xiamen PowerWay) diced into 5 × 5 mm chips (see Supporting Information section 1 for details). Arrays of hemispherical SILs with nominal radius 5 *μ*m were fabricated on these chips using the grayscale hard-mask lithography process set out in our previous work.^26^

The laser writing process for generating quantum defects in the center of prefabricated SILs in 4H-SiC is illustrated in Figure 1a. The laser writing was performed using a regeneratively-amplified Ti:Sapphire laser source, delivering 250 fs pulses at a wavelength of 790 nm and a repetition rate of 1 kHz (shown in Figure S1 and S2 in Supporting Information Section 2). The laser was focused through a high-NA oil immersion objective lens (Olympus 60×, 1.4 NA).

**Figure 1:**
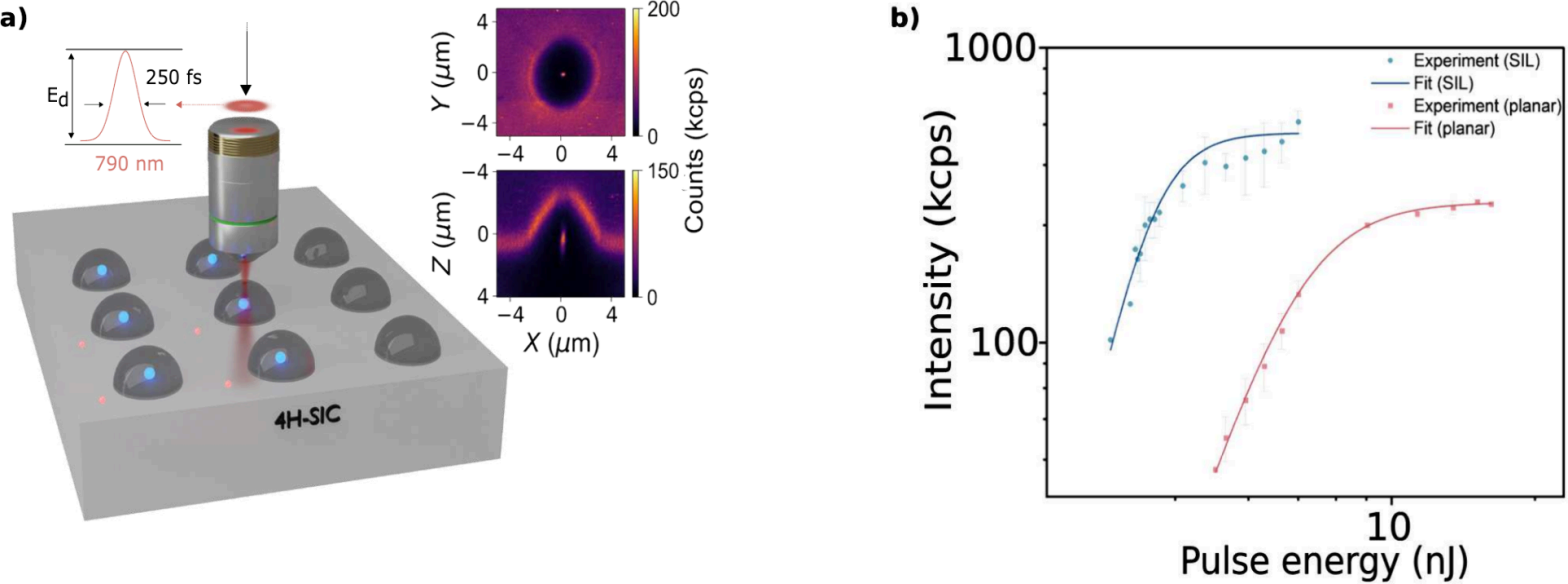
(a) Schematic illustration of the formation of V_Si_ centers in 4H-SiC via femtosecond laser writing using a single 250 fs FWHM pulse at 790 nm. Writing through the bulk (planar) interface results in lower efficiency compared to direct writing within SIL structures, as indicated by the contrast between dim red and bright blue spots, respectively. The inset presents photoluminescence (PL) maps of a defect ensemble written inside a SIL. The top panel shows an XY scan, and the bottom panel shows an XZ scan, demonstrating the precise spatial registration of laser-written defects in both axial and lateral dimensions relative to the SIL position. (b) Plot of the intensity of photoluminescence observed in laser-written defects as a function of writing pulse energy for bulk (red) and SIL (blue) interfaces, showing the effect of the SIL in reducing the waist of the focused beam and enhancing the local intensity of the pulse, resulting in an approximately 5-fold reduction in the required writing power for observing single emitters and higher light extraction efficiency. The experimental data were fitted using the saturation model described in eq 2.

A single femtosecond laser pulse with adjustable pulse energy was focused within the 4H-SiC sample. The degree of induced lattice damage can be controlled by varying the pulse energy, both when writing in the planar region and within SILs. For planar interfaces (red spots in Figure 1a), the focal depth was set to 5 *μ*m, matching the radius of the hemispherical monolithic SILs. Focusing into high-index materials like 4H-SiC induces strong spherical aberrations, which were corrected using a spatial light modulator (SLM).

During writing, the laser was focused through the SIL along its central axis at a depth corresponding to the sample's planar surface (blue spots in Figure 1a). This adjustment aimed to enable precise defect placement with minimal lattice perturbation and ensure optimal axial alignment between the SIL focus and laser-induced defects.

The laser writing process was initially characterized by photoluminescence (PL) under 532 nm CW optical excitation (1 mW) in a home-built confocal microscope with a 600–800 nm collection window (details discussed in Supporting Information section 2). In both SIL and planar configurations, the PL intensity increases with higher pulse energy, as shown in Figure 1b. For each pulse energy, data were collected from five spatially-separated sites (in the SIL case, 5 different neighboring SILs) written in a single column. All data points represent the arithmetic mean of intensities measured across these five equivalent fabrication points. Saturating behavior is observed in both cases, where the PL intensities detected from defect centers stabilize with respect to the writing power in the high energy regime.

Notably, significantly lower pulse energies are required for laser writing through the SIL compared to the planar interface, evidenced by the lower pulse energy range of the blue (SIL) curve in Figure 1b (1.4–4.0 nJ) relative to the red (planar interface) curve (4.0–20 nJ). This highlights the additional focusing effect provided by the SIL. The larger error bars in PL counts through the SIL interface arises from minor deviations in SIL geometry and alignment uncertainties during focusing the writing laser, both of which introduce aberrations to the incident wavefront and associated variation in focal intensity. Furthermore, the saturation count rate for the SIL interface reaches 600 kcps, compared to 300 kcps for the planar interface, which indicates the light extraction efficiency achieved through the SIL interface is also enhanced.

To elucidate the underlying mechanisms of laser-induced defect generation, we consider established models for ultrafast laser interactions in transparent materials, which typically involve multiphoton ionization (MPI), tunneling ionization (Zener breakdown), and avalanche ionization.^67^ As a starting point, we adopt the framework developed for the creation of GR1 centers (neutral carbon vacancies) in diamond,^68^ which shares key similarities with the processes in 4H-SiC. The 250 fs laser pulses employed in this work are significantly shorter than the characteristic timescales of thermal diffusion (nanoseconds),^69^ allowing us to neglect thermal effects and focus on nonthermal ionization dynamics. The relative contributions of MPI and tunneling ionization are governed by the Keldysh parameter,^67^ with MPI dominating when the laser intensity satisfies the condition:

1 $$I<\frac{mcn\epsilon_{0}E_{g}\omega^{2}}{e^{2}}$$

where *m* = 0.37*m*_e_ = 3.370 × 10^–31^ kg is the effective mass of lattice electrons, *n* = 2.6 and *E*_*g*_ = 3.23 eV are the refractive index and bandgap of 4H-SiC, and *ω* = 2.4 × 10^15^ Hz is the frequency of the photons. Substituting these values, the intensity threshold is calculated as *I* < 1.38 × 10^17^ W/m^2^. The pulse energy *E* is then determined as *E* = *I* × *π*× (beam waist)^2^ × (pulse duration), where for the planar interface the beam waist is 350 nm and pulse duration is 250 fs. Substituting these parameters, *E*_planar_ ≈ 16.0 nJ. For the SIL interface, the effective NA increases to ∼2.6, which reduces the beam waist to 190 nm, and the pulse energy is then calculated to be *E*_SIL_ ≈ 3 nJ. At very high pulse energies, MPI loses dominance, resulting in a deviation from the expected power-law scaling, as shown in Figure 1b. This potentially leads to lattice breakdown which is associated with the onset of broadband PL emission and a saturation plateau in the photoluminescence count rate, as observed in Figure 1b. Assuming that the PL intensity relates to the number of defects generated by the laser pulse; we fit this saturation model to the PL intensity *vs.* pulse energy data in Figure 1b (solid curves) to a saturation model:

2 $$I_{PL}(E)=\frac{aE^{n}}{1+kE^{n}}$$

where *a* is the amplitude coefficient, *n* is the power law exponent, and *k* is the saturation parameter (in slight contrast to the model presented in ref 68 for the negatively charged carbon vacancy center in diamond). Fitting for the SIL interface yields *n* = 5.75 ± 0.15 and *k* = (12.5 ± 1.26) × 10^–3^, while for the planar interface, *n* = 3.67 ± 0.15 and *k* = (1.83 ± 0.43) × 10^–3^. The enhanced nonlinearity in the SIL compared to the planar interface may arise from a stronger Zener breakdown contribution at lower pulse energies. The defect generation process involves photon energies that are multiples of the 790 nm laser photon energy. For the planar interface, this results in an energy of 5.8 ± 0.24 eV, while for the SIL interface, it yields 9.1 ± 0.24 eV. These energies exceed the SiC bandgap, enabling the generation of hot charge carriers rather than direct lattice disruption, given the nonpolar covalent bonding in SiC. Instead, multiphoton absorption excites carriers which transfer energy to the lattice, leading to defect formation. This carrier-mediated mechanism resembles Frenkel defect formation in diamond,^70^ highlighting a shared pathway for laser-induced structural modification in wide-bandgap materials.

PL measurements were performed at room temperature using a 780 nm laser, with 800 nm dichroic and shortpass filters (Thorlabs FES800) placed in the excitation arm to suppress residual laser light, as described in our previous work.^26^ By spatially mapping this PL emission over laser-written SILs, we could identify laser-induced defects localized near the center of SILs (Figure 2).

**Figure 2:**
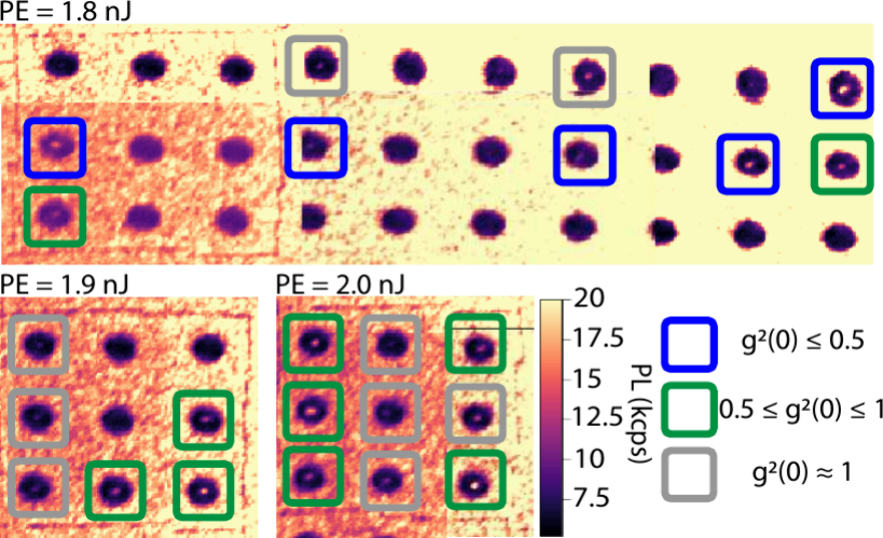
PL maps of regions where SILs were written with single laser pulse energy (PE) range to create single V_Si_ defects. This is shown by PE = 1.8 nJ, which has a statistically significant number of single photon sources distributed across the 30 SILs. Poissonian distributions in emitter creation are observed, progressing from 0.35 for PE = 1.8 nJ to 1.0 for PE = 2.0 nJ. When PE = 1.8 nJ, we also observe a higher yield of single color centers, namely 5 out of the 9 emitter spots observed in this region. This results in an expectation value of ≈0.35 as, out of the 9 SILs where a photoluminescent spot was created, 5 are confirmed as single photon sources. The other 21 SILs do not feature any photoluminescence.

For each SIL where a laser-written spot was detected, we measured the excitation power and brightness at which the emission saturates (*power saturation* measurement) and the normalized depth of the second-order correlation function at zero delay (*g* ^(2)^(0)), using low-jitter (∼40 ps) superconducting nanowire single-photon detectors (Single Quantum EOS). There is noticeable background luminescence from the SiC/air interface but not within the SIL itself. This agrees with previous studies in 4H-SiC that report intrinsic defects related to native surface oxide.^59^

Stochastic distributions of emitting defects consistent with Poissonian statistics could be detected across the written region, with the number and brightness of defects increasing with increasing laser-writing power. To minimize the creation of multiple emitters, writing is ideally performed in the regime where the Poissonian expectation value of creating any emitters is much less than one (⟨*n*⟩ ≪ 1). For example, for an expectation value of ⟨*n*⟩ = 0.1, the probability of creating a single emitter is *P*(1)_⟨*n*⟩=0.1_ = 9%, while the probability of creating two or more emitters is *P*(>1)_⟨*n*⟩=0.1_ = 0.4%. So, even for such a low yield, two emitters would be created approximately 5% of the time. In the case of the region with laser writing pulse energy PE = 1.8 nJ shown in Figure 2, 9 written defects occur within a set of 30 SILs, including five confirmed single photon emitters and two defects consistent with two emitters, leading to a Poissonian expectation value of ⟨*n*⟩_PE=1.8 nJ_ ≈ 0.35.

The resulting values are reported in Table 1; we show the second-order correlation results before and after background subtraction. Fabrication using laser pulse energy 1.8 nJ created the largest number of emitters with *g* ^(2)^(0) < 0.5; see Figure 2. The SIL ID in Table 1 denotes its grid location on the sample. We also show photoluminescence counts at saturation for each emitter.

**Table 1:** Statistics of Color Centers Generated in SILs via Femtosecond Laser Writing^a^

**SIL ID**	**PE (nJ)**	**PL**_sat_ **(kcps)**	***g*** ^**(2)**^ **(0)** **(raw)**	**g**^(2)^(0) **(bkg subtr)**
T30	1.9	38.1 ± 0.5	0.34 ± 0.02	0.27 ± 0.11
X18	1.8	36.0 ± 0.6	0.54 ± 0.05	0.45 ± 0.05
X23	1.8	29.0 ± 1.7	0.41 ± 0.04	0.21 ± 0.04
X26	1.8	45.7 ± 2.3	0.44 ± 0.07	0.27 ± 0.11
AA32	1.6	23.3 ± 1.8	0.55 ± 0.06	0.43 ± 0.06
Y17	1.8	32.3 ± 0.8	0.61 ± 0.04	0.40 ± 0.05
X20	1.8	38.3 ± 1.4	0.62 ± 0.05	0.54 ± 0.06

a SIL ID: column and row label of SIL with respect to one corner of the array. PE (nJ): writing laser pulse energy. PL_sat_: saturation photoluminescence counts of each emitter. *g* ^(2)^(0): normalized second-order correlation at zero delay without background subtraction (raw) and with background subtraction applied (bck subtr). After background subtraction, the laser-written defects are confirmed to be single photon emitters, occurring most frequently at PE = 1.8 nJ. SIL ID T30 is the emitter characterized in Figure 3.

We classify each PL spot based on its *g* ^(2)^(0) value to be either a single emitter (*g* ^(2)^(0) ≤ 0.5), multiple emitters 0.5 < *g* ^(2)^(0) < 1, or as a PL spot not in the single-emitter regime *g* ^(2)^(0) = 1.

Throughout several months of measurements, we have seen no evidence of photobleaching, and the PL emission for all examined color centers has remained stable and reproducible.

We examined the PL spectra of generated quantum emitters at low temperature (*T* = 4 K, Montana s100 Cryostation) to identify characteristic zero-phonon lines (ZPLs). The emitters were excited with a 780 nm CW laser, and the PL spectra were measured with a grating spectrometer (OceanOptics QE Pro)). For further information on the setup and measured spectra, see Supporting Information section 5.

Remarkably, we observe a wide spread of ZPL wavelengths in the spectral region 858–985 nm. Out of 39 emission defects examined, 6 have spectral lines that can be classified as silicon-vacancy (V_Si_) centers; these were 1 V_1_′ center (ZPL 858 nm), 4 V_1_ centers (h-site, ZPL 861 nm), and 1 V_2_ center (k-site, ZPL 916 nm). The remaining defects exhibit ZPLs at different wavelengths which do not appear to match other unidentified lines previously reported in this spectral range^71–73^ but fall within the theoretically-predicted range of V_Si_ centers modified by nearby carbon anti-sites^74^ and experimentally-observed range for V_Si_ centers in etched membranes.^75^

We further performed optically-detected magnetic resonance (ODMR) measurements at room temperature, in the frequency ranges associated with V_1_ (zero-field splitting 4 MHz) and V_2_ (zero-field splitting 70 MHz) centers. For as-written emission defects, no ODMR signals were observed in these ranges. However, after annealing the sample at 600°C for 30 minutes in vacuum (4 × 10^–5^ mbar), a process known to improve V_Si_ yield, we observed an ODMR signal associated with V_2_ for one SIL (of 12 measured), though with a large linewidth of about 20 MHz (see Supporting Information section 6 Figure S6).

One possible explanation for the lack of ODMR signal might be that the femtosecond laser creates other defects that are not optically active, in addition to the V_Si_. For example, carbon atoms feature much lower displacement energy than silicon atoms in SiC,^76–79^ so that the creation of carbon vacancies (V_C_) is favored. The presence of charge traps related to carbon vacancies and other defects^64,65,80^ is expected to create electromagnetic noise that may broaden observed optical and magnetic resonance linewidths. Further experiments, beyond the scope of this work, are needed to clarify the physics of femtosecond laser generation of point defects and to optimize laser writing parameters for improving the quality of generated quantum emitters.

In Figure 3, the key properties are given of a single V_Si_ center generated in SIL T30 with a laser writing pulse energy PE = 1.9 nJ. The photoluminescence map (Figure 3a) shows a spot centered well with respect to the SIL profile (dark circle). The photoluminescent background outside of the SIL region corresponds to surface defects on the etched surface of the SiC, postulated to arise at the SiC-surface oxide interface.^29,59,81–83^ Spectroscopy of the emitted light at cryogenic temperatures (*T* = 4 K) yields a ZPL at 861 nm, consistent with a V_1_-type V_Si_ center. Furthermore, even without background subtraction, the normalized second-order correlation at zero delay *g* ^(2)^(0) = 0.34 ± 0.018, confirms that the emission comes from one single photon emitter. Taking power saturation measurements of the single V_Si_ center in SIL T30 and a single electron-irradiated V_Si_ center beneath a planar interface (Figure 3, blue and green, respectively), the performance of the SIL-registered emitter could be benchmarked. Following our previous work,^26^ we determine an optical collection efficiency enhancement factor of 4.5 (PL_sat,SIL_ = 38800 ± 500 vs PL_sat,bulk_ = 8600 ± 600) and a power intensification factor of 9.1 (*I*_sat,SIL_ = 0.181 ± 0.014 and *I*_sat,bulk_ = 1.64 ± 0.21), consistent with the upper levels of performance observed for single V_Si_ centers generated randomly throughout the SIL by electron irradiation.

**Figure 3:**
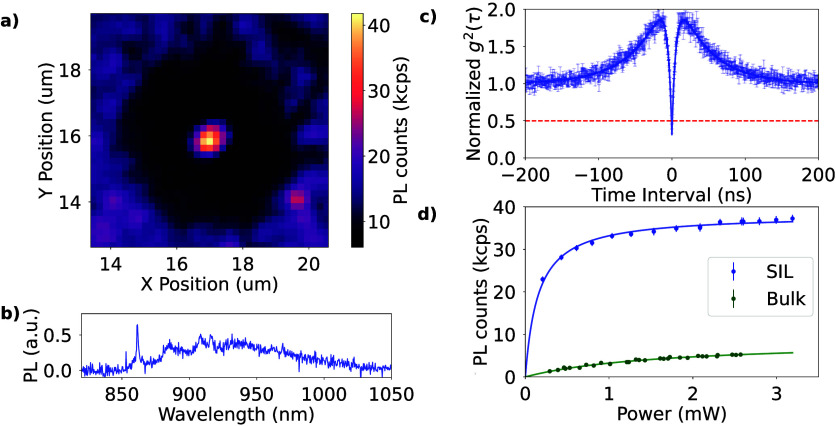
Characterization of a single V_Si_ written in SIL T30 with PE = 1.9 nJ. (a) Photoluminescence map taken at an excitation power of 4.2 mW. Note the background observed on the outskirts of the map is associated with SiC interfacial emitters^59^ and does not occur within the SiC crystal. (b) An optical spectrum was taken at low temperature (4 K), showing a zero-phonon line at 861 nm characteristic of a V_Si_ at a h lattice site. (c) Normalized autocorrelation plot of light from the emitter without background subtraction. The depth at zero time delay *g*_2_(0) = 0.35 ± 0.015 confirms a single V_Si_ has been created. (d) Power saturation curve of emitter T30 (blue), with a representative curve of a single emitter under a planar interface (green) for reference. The brightness of the emitter is enhanced by 4.47 times, and excitation power is intensified by a factor 9.2.

Laser writing through the SIL interface was performed by microscope-aided alignment of the laser focus to the center of the SIL at a depth of 5 *μ*m, equivalent to the height of the lens structure. Contributions to the final emitter misalignment with respect to this target could arise both from the random probability of generating a defect within the volume of highest writing intensity and the error in writing laser alignment.

To quantify these distributions, spatial PL maps of SILs with emitter spots were characterized using image analysis software (ImageJ) to extract the center of each SIL and emitter spot (see Supporting Information section 3). The absolute displacements of measured emitters from their collective mean position are given in Figure 4a. The statistics follow a Rayleigh distribution with scale parameter *σ* = 0.14 *μ*m (dashed line).

**Figure 4:**
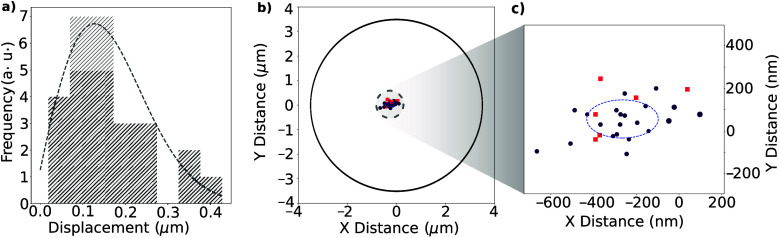
Quantification of emitter distribution within SILs through registered laser writing. (a) Histogram of absolute displacement of the center of each observed emitter spot from the center of the SIL, determined using spatial PL maps. (b) Schematic of relative distributions in position space, with a SIL outline of radius 3.5 *μ*m as a guide for PL profiles. (c) Zoomed-in position map showing the distribution of emitter positions more clearly, with the dashed line denoting the mean displacement obtained from fitting the histogram data. Points in purple are registered positions with *g* ^2^(*τ*) > 0.5; the red squares are single defects that are found in the Table 1.

When the emitters’ absolute positions with respect to the SIL are plotted (Figure 4b), clustering near the SIL center is evident, especially compared to emitter generation through electron irradiation in similar structures.^26^ Focusing on the SIL center (Figure 4 c), we find that the standard deviation of positions in the *x*-direction (±170 nm) is higher than in the *y*-direction (±90 nm). This is possibly due to the raster protocol for laser writing, where writing proceeds along all columns in a row before moving to the next row, so that more steps are taken in *x* than in *y*.

When aligning the writing laser to SILs, systematic offsets in positioning or beam angle can lead to the mean defect position deviating from the structure’s center. This is observed in Figure 4c, where the mean emitter position is offset by 260 nm in *x* and 60 nm in *y* from the SIL center.

In this work, we have demonstrated marker-free registration of single quantum emitters at the center of SILs by femtosecond laser writing. The SILs lower the laser dose required to generate emitters and aid in positioning them near the center of the structure, with an accuracy of 60 ± 90 nm in the *y*-direction and 260 ± 170 nm in the *x*-direction. Our approach, based on direct alignment of the laser-writing beam focus to the center of the SIL, achieves sub-diffraction-limit placement accuracy within the SIL to maximize optical collection enhancement. In future applications, better positioning (potentially <100 nm) could be achieved through use of alignment markers.

There remains an open question regarding the nature and diversity of created emitters and their performance for quantum technology applications. In our post-annealing study, controlled thermal treatment revealed opportunities to enhance the spin coherence of the generated defects; however, the yield of high-coherence centers remained low. Future investigations could systematically identify generated defect species and map their optical and spin-coherence properties as functions of laser irradiation parameters and thermal annealing protocols. Furthermore, deterministic generation of V_Si_ centers can be further pursued by integrating laser-annealing sequences with in-situ photoluminescence monitoring.

## Supplementary Material



## Notes

Experimental details of ODMR measurements, optical spectra, and XZ profiles, descriptions of the experimental setups, fitting procedure details, and sample preparation methods (PDF)
