# Supplementary material for: Scalable Registration of Single Quantum Emitters within Solid Immersion Lenses through Femtosecond Laser Writing
Source: Nano Lett. 2025 Jun 10;25(30):11528–35. doi: 10.1021/acs.nanolett.5c01325 (PMC12314893; doi:10.1021/acs.nanolett.5c01325)
Supplement: Supplementary file 1 [file nl5c01325_si_001.pdf]

Scalable registration of single quantum emitters within solid immersion lenses through femtosecond laser writing

Alexander R. Jones,[1,*] Xingrui Cheng,[2,3,*] Shravan Kumar Parthasarathy,[4,5] Muhammad Junaid Arshad,[1] Pasquale Cilibrizzi,[1] Roland Nagy,[4] Patrick Salter,[2] Jason Smith,[3] Cristian Bonato,[1,†] and Christiaan Bekker[1,‡]

[1]*Institute of Photonics and Quantum Sciences, SUPA, Heriot-Watt University, Edinburgh EH14 4AS, UK*
[2]*Department of Engineering Science, University of Oxford, Parks Road, Oxford OX1 3PJ, UK*
[3]*Department of Materials, University of Oxford, Parks Road, Oxford OX1 3PH, UK*
[4]*Institute of Applied Quantum Technologies, Friedrich-Alexander-University Erlangen-Nrnberg, 91052 Erlangen, Germany*
[5]*Fraunhofer Institute for Integrated Systems and Device Technology (IISB), 91058 Erlangen, Germany*

SUPPLEMENTARY INFORMATION

S1. Sample Preparation

The experiment was conducted using commercial 4H-SiC material (Xiamen PowerWay©) diced into 5×5 mm chips.With substrate and epilayer thickness 500 μm and 15 μm respectively, and residual n-doping level of $< 1 \times 10^{14}$ cm^{-3}. The material was diced into 5×5mm chips. Arrays of hemispherical SILs with nominal radius 5μm were fabricated on these chips using the grayscale hard-mask lithography process set out in our previous work [1].

S2. Laser writing system

The laser writing system employed in this work is consistent with that described in a previous study [2]. The system comprises a Spectra Physics Mai Tai laser and a Spectra Physics Solstice amplifier, operating at a wavelength of 790 nm with a maximum repetition rate of 1 kHz. Pulse energy is regulated via a $\lambda/2$ waveplate in combination with a Glan-laser polarizer. The beam is subsequently expanded before reaching the spatial light modulator (SLM, Hamamatsu Photonics X10468-02). A dichroic mirror directs the fabrication laser toward the sample while transmitting both the excitation and PL signals from an integrated room-temperature confocal system, featuring a 532 nm CW laser as the excitation source. Laser fabrication and PL characterization employed an Olympus PlanApo 60 oil−immersion objective (NA = 1.4) with immersion oil of refractive index of n = 1.5. Emission under 532 nm excitation was spectrally filtered to the 600800 nm window using a bandpass filter and a notch filter. Schematics of the laser writing system and confocal microscope are provided in Fig. S1 and Fig. S2, respectively.

S3. Description of the circle-fitting procedure

Here we describe the procedure to assess the position of the created emitter with respect to the SIL, based on PL maps with a stepsize of 0.13 μm. First of all, we determine the SIL circular edge to retrieve its center. We used a data analysis application (ImageJ) to determine the SIL circular edges, using three different methods to verify the reproducibility of the outcomes. First, we use the *fit circle* tool to fit an exact circle around the SIL; second, we use the *ellipse tool* to fit precisely the SIL edge. Lastly, we used the profile tool to measure the changes in PL, which are expected to be high at the SIL edge as the surface of SIL itself fluoresces. We then fit a Gaussian to each of these PL peaks to pinpoint a precise position for the edges. Fig. S3 shows a comparison between the three methods for the same SIL. The ellipse method in orange, the circle method in yellow and the blue lines indicate where the profiles would be taken to find the SIL edge as the PL counts rise relative to the background within the SIL.

Similarly, we compare results from two methods to determine the emitter position. First, we use the fit circle tool in ImageJ, we fit a circle around the emitter confocal spot, and retrieve its center. Second, we fit a Gaussian to the

* Both authors contributed equally to this work.
† c.bonato@hw.ac.uk
‡ c.bekker@hw.ac.uk

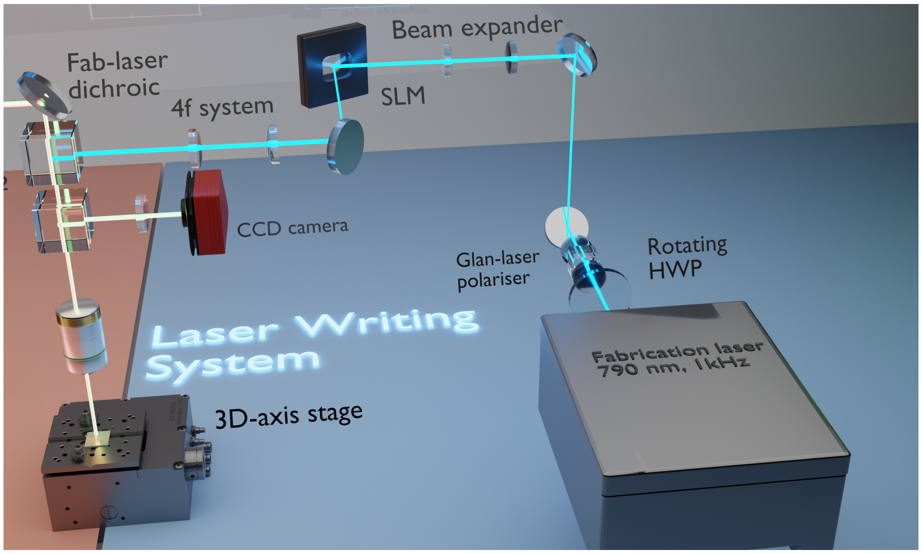

Supplementary Figure S1. Schematic view of the Laser Writing System, including the fabrication laser for both kHz and MHz, SLM, ($\lambda/2$) waveplate, Glan-laser polarizer, CCD camera, 4f system, and translation stages for sample positioning.

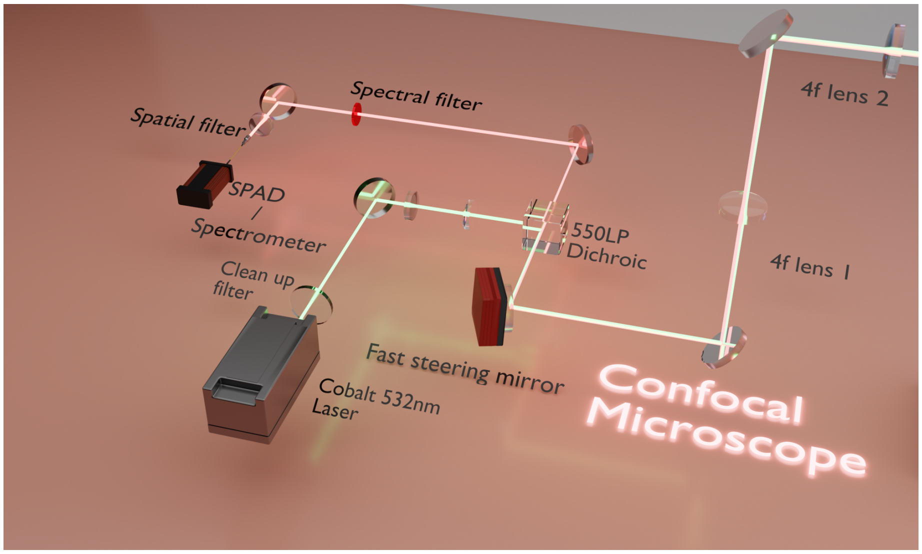

Supplementary Figure S2. Schematic view of the confocal module, including the excitation laser, beam rastering module with FSM and 4f configuration relay lenses, and the collection system for SPAD or spectrometer detection.

emitter confocal spot. Fig. S3 shows the typical positions for these methods, where the blue lines again show the profile positions and the green shows the circle that would fit around the defect to determine the central point to be taken as the defect position.

We used the results for all SILs that generated a spot at 1.6 nJ writing power to benchmark the methods. The average SIL center using the ellipse and fitted circle methods were the same as 0.55μm, $0.29\,\mu$m. The average calculated position relative to the SIL center using the profile method was 250nm $\pm\,0.09$ in the x, and 78nm $\pm\,0.04$ in the y. The average calculated position for the defect relative to the SIL center using the fitted circle's method was 190nm $\pm\,0.11$ in x and 96nm $\pm\,0.05$ in the y-direction. Due to the defects being within the lens, we performed a correction to account for the magnification effects on the measured distance between the center of the spot and the SIL.

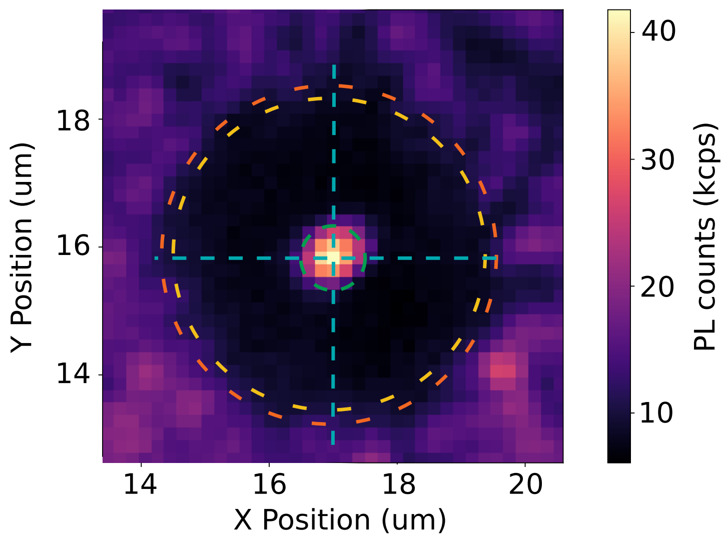

Supplementary Figure S3. Using SIL T30 from Fig. 3, we illustrate how we identified the positions of the registered defect centers within the SIL. The blue and green lines indicate the profile and circle methods, respectively. These were used to find the defect positions. The yellow, orange, and blue lines illustrate the circle, ellipse, and profile methods, respectively. These were used to find the SIL centers.

S4. XZ profiles in SILs

Fig. S4 presents a series of XY and XZ 2D PL images for defects fabricated with high pulse energy of 3.5 nJ (a) and low pulse energy of 2 nJ (b). These PL images indicate that the defect centers are registered near the center of the SIL in both lateral and axial directions, aligning well with the SIL's focus. The elongated point spread function (PSF) observed in the axial PL profiles is attributed to the increased Abbe resolution in confocal microscopy. Quantitatively, the lateral and axial resolutions extracted from low pulse energy fabricated defect (110 nm and 170 nm, respectively) align with diffraction-limited expectations (100 nm lateral, 160 nm axial). In contrast, defect fabricated with higher pulse energy exhibits broadened emisssion profile (210 nm lateral and 985 nm axial, respectively), attributed to increased lattice damage induced by higher pulse energy that disrupts the point-source approximation.

S5. Optical Spectra of Emitters

To verify the characteristics of the quantum emitters produced, their photoluminescence (PL) spectrum was analyzed at low temperatures using a cryostat set to 4K (Montana s100 Cryostation) in combination with a custom confocal setup, as detailed by Cilibrizzi et al. [3]. The emitters were illuminated with a continuous wave (CW) laser at 780 nm, and their PL spectra were recorded with a grating spectrometer (OceanOptics QE Pro) with a 800nm longpass filter. The spectra obtained are displayed in Supplementary Figure S3.

The analysis uncovered a broad distribution of zero-phonon lines (ZPL) within the 858-985 nm spectral region. Out of 39 examined defect centers, six exhibited spectral lines typical of silicon-vacancy (V_{Si}) centers: one V_1' center (ZPL at 858 nm), four V_1 centres (h-site, ZPL at 861 nm), and one V_2 center (k-site, ZPL at 916 nm). Other defect centers showed ZPLs at various wavelengths within the 858-985 nm range, which do not correspond to previously reported unidentified lines in this range but align with the theoretically predicted range of V_{Si} centers modified by nearby carbon anti-sites.

The broad line widths observed in the PL spectra and the lack of an ODMR signal might be attributed to the femtosecond laser's creation of additional non-optically active defects alongside the V_{Si}.

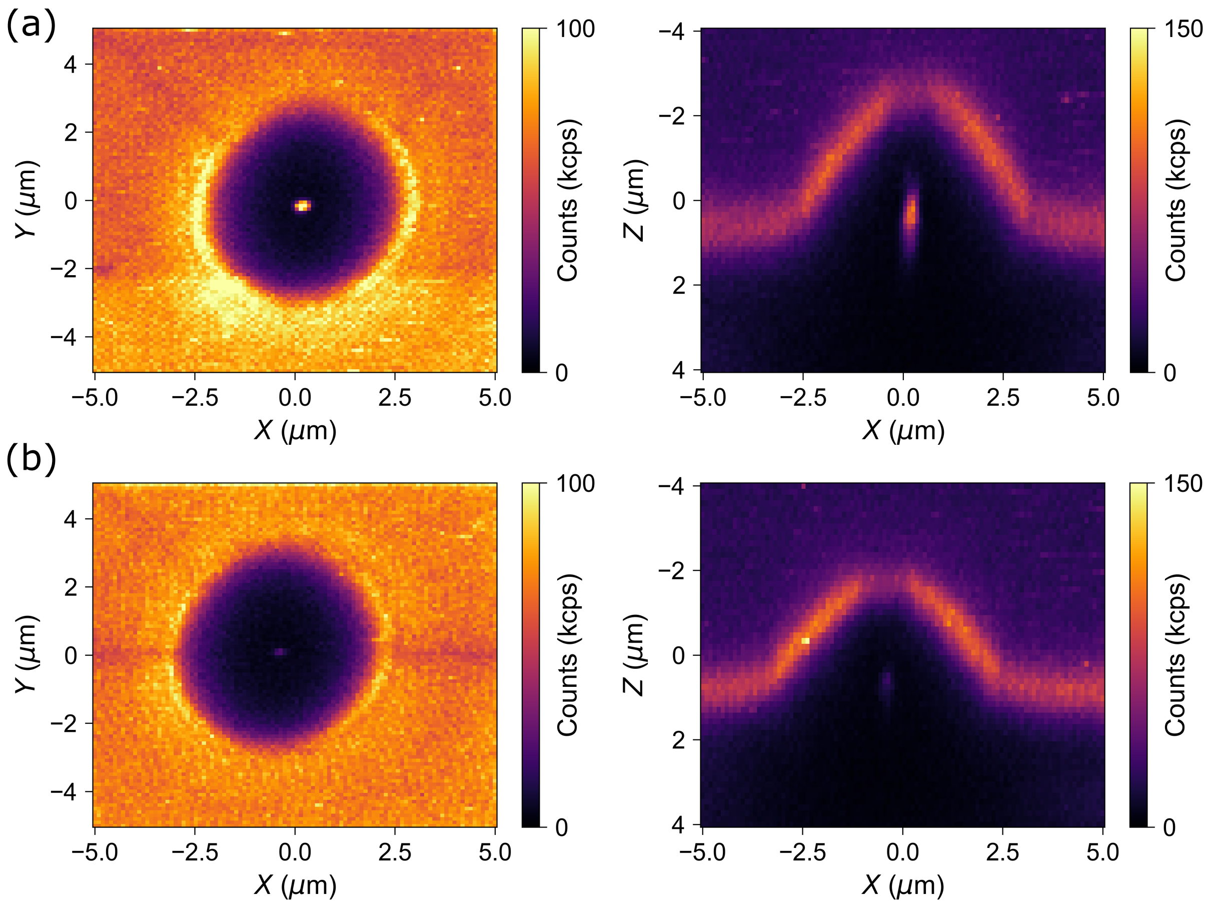

Supplementary Figure S4. XY and XZ 2D PL images of defect centers created with (a) high and (b) low pulse energies. High pulse energy results in an extended emission region due to lattice damage, while low pulse energy exhibits diffraction-limited behavior. Defect centers are aligned with the SIL focus, and the elongated XZ PSF reflects increased Abbe resolution.

S6. ODMR after Anneal

An anneal at 600 degrees was conducted for 30 minutes on the sample in vacuum at 4×10^{-5} mbar to investigate the stability of the observed defects, and whether they could be converted to silicon vacancies. We re-characterised 5 SILs, which originally showed no ODMR. One evidenced an ODMR peak after annealing, with 0.48% contrast and 22.36 MHz linewidth (Fig. S6).

The ODMR measurement is performed by focusing an off-resonant cw-laser (730 nm) on the a-plane side of the 4H-SiC sample inside the cryostat (Attocube Attodry 800) at 4K with a high-NA objective (Zeiss Epiplan-Neofluar 100x, NA 0.9). The spin state manipulation of the color center is orchestrated with the help of an Arbitrary Waveform Generator (QM-OPX) and a copper wire (50 μm diameter) running on top of the sample. The AWG feeds a radio-frequency signal with a power of -27dBm, amplified by a 44 dB amplifier (LZY-22+ Mini-circuits) and fed to the microwave antenna wire running over the sample. The manipulation of the spin states is realized once the frequency of microwave matches with the zero field splitting of ground state spin (70 MHz for a V_2 color center at $B_0 = 0$G). The difference in spin state population while sweeping the MW frequency is seen through the photoluminescence emitted by the V_2 V_{Si} center which is filtered using a dichroic mirror (Semrock FF925-Di01) and along pass filter (FELH950 Thorlabs). The emission is readout through a super conducting nanowire single photon detector (Single Quantum).

The filtering during spectral acquisition was carried out using a SEMROCK long-pass filter in the transmission direction, oriented at approximately 50° to achieve an effective cutoff near 900 nm. This configuration was chosen to remove stray light from the 730 nm excitation laser, which has a mild band extending to 1000 nm. The 900 nm filter allows transmission of zero-phonon lines (ZPLs), including the V_2 center at 916 nm. To enhance clarity for the reader, cosmic ray artefacts were removed during post-processing.

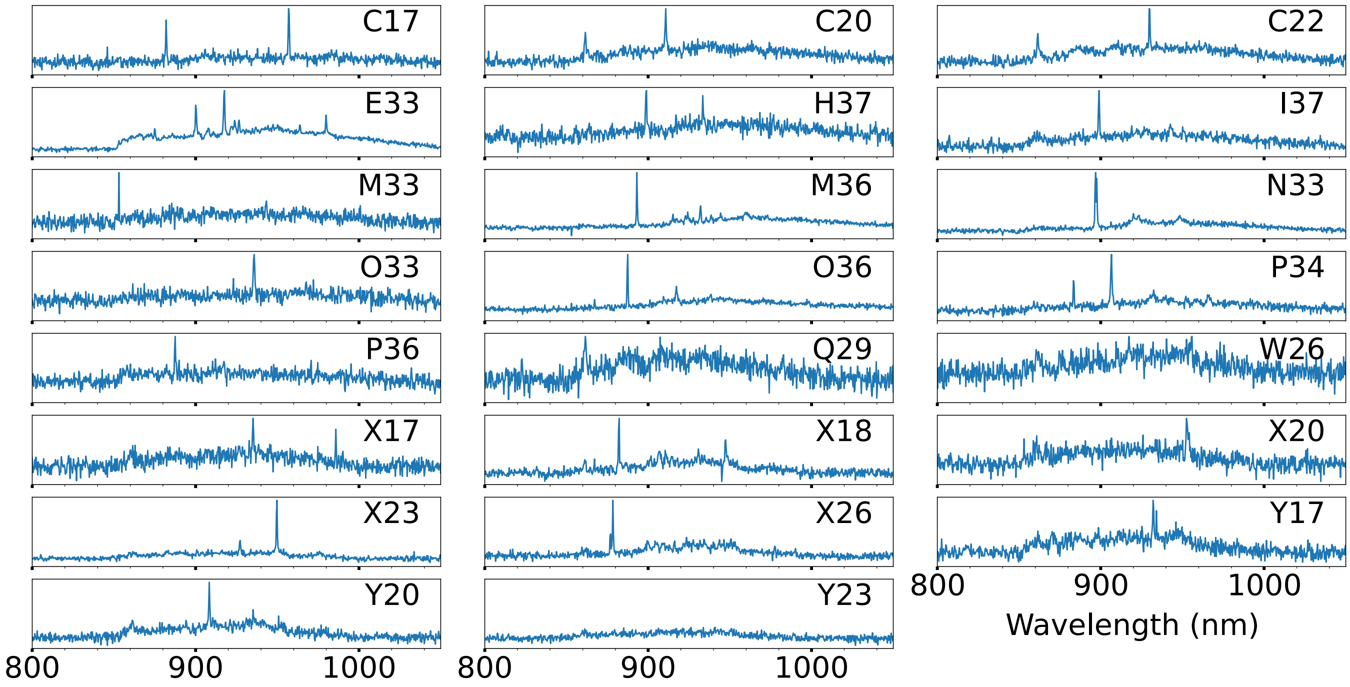

Supplementary Figure S5. Optical spectra were taken at low temperatures using the setup previously discussed in the Results Section for laser-written defects in SIL structures. They show a range of zero-phonon lines consistent with the predicted region for modified V_{Si} centers. Labels correspond to SIL array position, as described in the main text. Zero-phonon lines are consistent with V_{Si} centers observed for SILs C20, C22, Q29, T30, O36, and Y20.

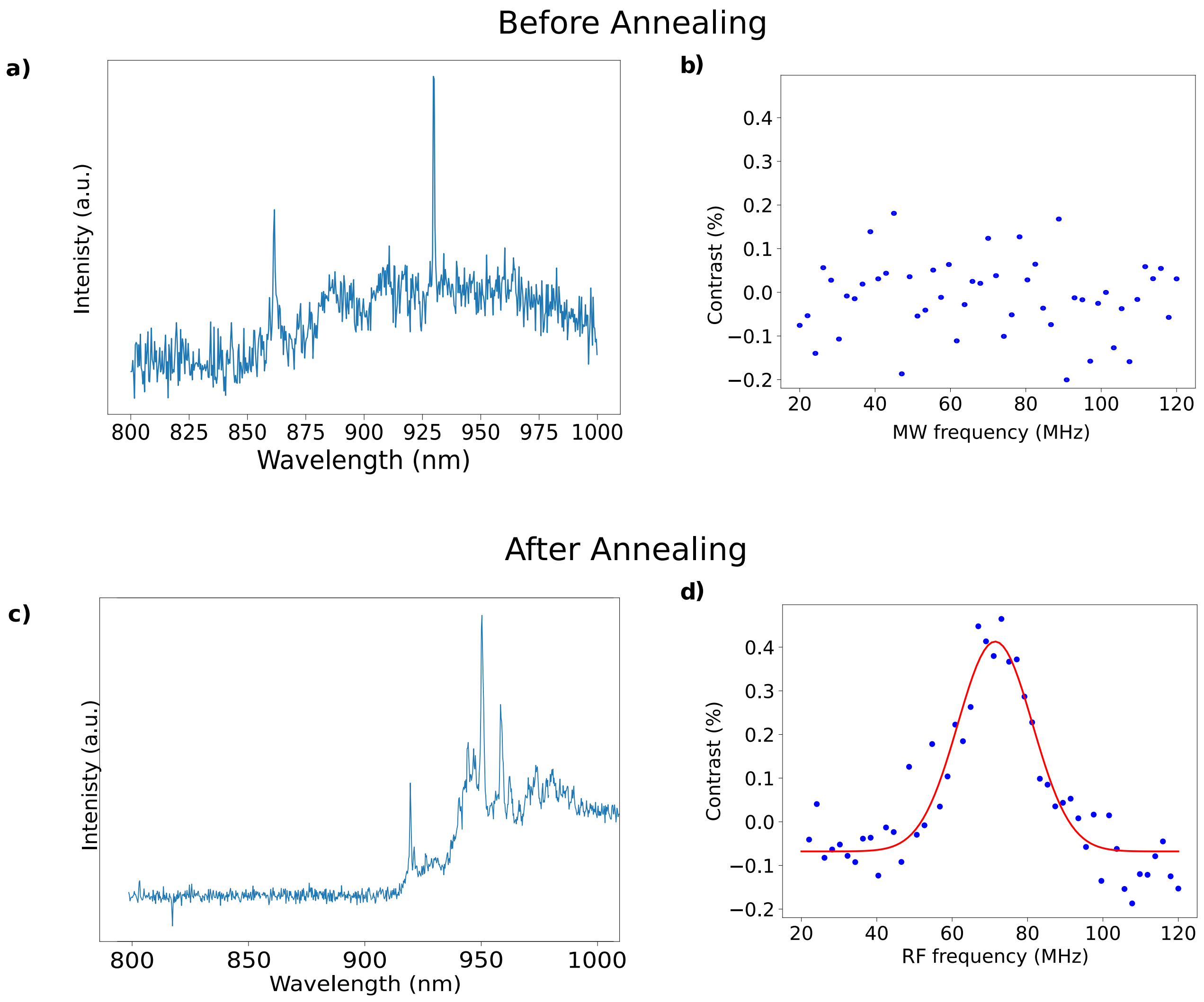

Supplementary Figure S6. This ODMR was conducted at room temperature on SIL C22 using a 730 nm later to excite the color center off resonantly while sweeping the microwave frequency (MW) from 20 MHz to 120 MHz. The spectrum is centered on 71.41 ± 0.6MHz and Full-width half-maximum at 22.36 ± 2.4MHz. The low-temperature spectra in a) is the same as previously shown in the SI section 5.

[1] C. Bekker, M. J. Arshad, P. Cilibrizzi, C. Nikolatos, P. Lomax, G. S. Wood, R. Cheung, W. Knolle, N. Ross, B. Gerardot, and C. Bonato, Applied Physics Letters **122**, 173507 (2023).
[2] Y.-C. Chen, P. S. Salter, M. Niethammer, M. Widmann, F. Kaiser, R. Nagy, N. Morioka, C. Babin, J. Erlekampf, P. Berwian, M. J. Booth, and J. Wrachtrup, Nano Letters **19**, 2377 (2019).
[3] P. Cilibrizzi, M. J. Arshad, B. Tissot, N. T. Son, I. G. Ivanov, T. Astner, P. Koller, M. Ghezellou, J. Ul-Hassan, D. White, C. Bekker, G. Burkard, M. Trupke, and C. Bonato, Nature Communications **14**, 8448 (2023).
